# On Modern Progress in Ophthalmic Medicine and Surgery

**Published:** 1894-02-24

**Authors:** Robert Brudenell Carter

**Affiliations:** Consulting Ophthalmic Surgeon to St. George's Hospital


### On Modern Progress in Ophthalmic Medicine and Surgery.

By Robert Brudenell Carter, F.R.C.S., Consulting Ophthalmic Surgeon to St. George's Hospital.

The case being decided to be one of glaucoma, the next question to be considered must be that of the operation most appropriate to its relief, and of the degree in which the performance of this operation should be regarded as urgent in point of time. Prior to the researches of Mr. Priestley Smith, and to his discovery of the part played by the periphery of the iris in obstructing access of fluid to the filtration area, the good effects of iridectomy were often attributed to the pervious character of the cicatrix of the external wound, and it was suggested that such a pervious cicatrix might be produced without touching the iris at all. Hence was devised the operation called sclerotomy, in which the surgeon carries a narrow cataract knife, with its cutting edge upwards, through the anterior chamber by puncture and counter-puncture, and then divides the firmer tissues of the sclero-corneal junction, but leaves a central bridge of conjunctiva uncut. If badly done, sclerotomy is often followed by considerable prolapse of iris into the incision, and by the formation of a thin prominent cicatrix; so that there is at least as much deformity of the pupil as after an iridectomy, and the cicatrix forms an uncomfortable prominence beneath the upper lid. If skilfully done, and if the eye is closed and dressed without pressure, the bridge of conjunctiva will allow the lips of the incision to fall a little apart, and the result will be a line of thin cicatricial tissue, of a manifestly pervious character, which, in certain cases, will provide sufficiently for the future transudation of superfluous intra-ocular fluid. Speaking generally, it may be said that sclerotomy is a less efficient operation than iridectomy, and that it should never be employed in acute or in advanced chronic cases. In the early stage of a chronic case it may be practised, and will often accomplish all that is required. When it fails to do this, it will at least be a temporary palliative, and it leaves iridectomy as a resource to be employed at any future time. Its advantages are that it is somewhat less severe than iridectomy as a procedure, that it never requires a general anæsthetic, and that, by leaving a normal circular pupil, it does not occasion the dispersion of light, and the consequent lowering of vision, which are sometimes consequences of iridectomy, even although the operation has manifestly prevented the occurrence of total blindness. None of these advantages are sufficient to be set against the more certain efficacy of iridectomy in the great majority of instances, although they afford the surgeon an alternative operation in some that must be carefully selected for the purpose.

With regard to urgency, an operation of some kind is always urgent. Herniotomy can never be more urgently required for the preservation of life than iridectomy sometimes is for the preservation of sight; and no surgeon is fully equipped for the duties of his profession unless he is prepared to perform either one or the other in case of need. But iridectomy, although very easy in a normal eye, with a good anterior chamber, and plenty of room for the entrance of the knife, is extremely difficult in many of the cases in which it is most required. The pupil may be dilated, and the iris pushed forward by the lens until the anterior chamber is almost abolished; while, the lens being still transparent, the precise position of its anterior surface cannot be localised with any certainty, and it may easily be wounded, and traumatic cataract produced, in making the external incision. Fortunately, in all but the "fulminating" cases, and generally in acute glaucoma as long as good perception of light is retained, it is possible to diminish the difficulties, and, at the same time, to relieve the symptoms, by the diligent instillation of a solution of eserine. An eserine wafer, or a drop of a solution of four grains to the ounce, applied every hour for five or six times, will generally produce some contraction of the pupil; and the consequent partial removal of the iris from the filtration area will relieve the extremity of the tension. In this way, by waiting a few hours, the surgeon will often find his patient inspirited by the commencement of improvement in vision, while, at the same time, the pupil will be comparatively small, and all but the centre of the lens will be covered by the iris, which will then form a visible background serving to protect the lens from accidental injury. If the pupil be sufficiently small, the point of the straight narrow knife need never enter the pupillary area, but can be carried quite above its upper boundary across the anterior chamber, and the cutting edge brought out much as in the ordinary operation for cataract. It is generally desirable to combine cocaine with the eserine, the former agent being always overpowered by the latter as far as the diameter of the pupil is concerned, but nevertheless exerting an important influence in diminishing the calibre of the blood vessels, and therefore in diminishing the tendency to hæmorrhage. In an eye of high tension, it is seldom possible to obtain sufficient absorption of cocaine to produce anæsthesia of the iris, although the external incision may usually be rendered painless. Hence, in acute glaucoma, as the proper excision of the iris is the most important step of the procedure, it is better to administer ether or chloroform, unless the patient is a person of fortitude, prepared, if necessary, to bear a certain amount of pain without unsteadiness. A sudden upward roll of the eye, at the moment when the iris is seized by forceps, may defeat the intentions of the most dexterous surgeon.

In order to accomplish the excision of the iris in the best way, and to remove the whole width of the membrane, the closed iris forceps should be carried into the anterior chamber a little to the right of the centre of the external incision, and advanced nearly to the edge of the pupil. They should then be suffered slightly to expand, so that a fold of iris rises between them and is grasped as they are again closed. The operator should wait two or three seconds before commencing traction, and should then draw out the fold of iris as completely as possible. He should divide it with the points of keen scissors at the right hand angle of the wound, tear it away from its attachments up to the left hand angle, and there cut it away with the scissors, using such gentle pressure of their blades against the globe as may push back the external tunics, and cause the iris itself to retract within the anterior chamber when the pressure is removed. Effused blood may be left to undergo absorption, and a simple dressing will complete the operation.

